# 1700 nm and 1800 nm band tunable thulium doped mode-locked fiber lasers

**DOI:** 10.1038/s41598-017-13200-x

**Published:** 2017-10-06

**Authors:** Siamak Dawazdah Emami, Mahdi Mozdoor Dashtabi, Hui Jing Lee, Atoosa Sadat Arabanian, Hairul Azhar Abdul Rashid

**Affiliations:** 1 Faculty of Engineering, Multimedia University, 63100 Cyberjaya, Selangor Malaysia; 2 Laser and Plasma Research Institute, Shahid Beheshti University, Evin, Tehran Iran; 3 Photonics Technology Research Group, Universiti Tenaga Nasional, 43000 Kajang, Selangor Malaysia

## Abstract

This paper presents short wavelength operation of tunable thulium-doped mode-locked lasers with sweep ranges of 1702 to 1764 nm and 1788 to 1831 nm. This operation is realized by a combination of the partial amplified spontaneous emission suppression method, the bidirectional pumping mechanism and the nonlinear polarization rotation (NPR) technique. Lasing at emission bands lower than the 1800 nm wavelength in thulium-doped fiber lasers is achieved using mode confinement loss in a specially designed photonic crystal fiber (PCF). The enlargement of the first outer ring air holes around the core region of the PCF attenuates emissions above the cut-off wavelength and dominates the active region. This amplified spontaneous emission (ASE) suppression using our presented PCF is applied to a mode-locked laser cavity and is demonstrated to be a simple and compact solution to widely tunable all-fiber lasers.

## Introduction

Interest in fiber lasers emitting in the wavelength range of 1700 to 2100 nm has been escalating in recent fiber laser related research. This eye-safe^1^ wavelength region has various applications, such as light detection and ranging (LIDAR) systems^2^, biomedical science^3^, optical sensing^4^, and industrial machining^5^. This attention is also related to the exploration of promising transmission capacity beyond the conventional 1550 nm region^6^. Recent studies focused on achieving optimum signal transmission in the range of 1700 to 1900 nm. For such a case, hollow-core photonics band-gap fibers (HC-PBGFs) are proposed^7,8^. These fibers are able to overcome the capacity limit of traditional systems due to their ultra-low non-linearity and near-vacuum latency. Additionally, 1700 nm is the optimum wavelength for brain related studies^9,10^ in terms of tissue penetration when both tissue scattering and absorption are considered. This spectral window is promising for multi-photon nonlinear optical imaging of the brain. The sensitivity and the penetration level at 1700 nm are also high for ultrahigh-resolution optical coherence tomography (UHR-OCT)^10^. Peak absorption is observed at approximately 1720 nm for human tissues with lipids where the water absorption is exceeded by the fat absorption. Such phenomena would contribute to the treatment of different types of skin where the tissues with lipids can be selectively targeted^9^.

The numerous applications at the 1700 to 2100 nm region have driven researchers to focus on thulium-doped fibers (TDFs)^7–15^. TDFs have recently been fabricated, which generate gain in a broad wavelength range. Such fibers successfully enhanced the operation of optical amplifiers, continuous-wave fiber lasers and super luminescent sources in the 1700–2100 nm range that peaks at approximately 1900 nm^6,16^. Although the invention of these fibers broadened the achievable emission region, lasing at a wide tunable range remains an important challenge. To achieve such a wide tunable range, the gain bandwidth of lasing emission has to be broadened, and the center wavelength has to be tunable^16,17^.

To shift the center-wavelength in fiber amplifiers, methods such as the formation of an artificial spectral filter comprising a tapered fiber^18^, a fiber Bragg Grating (FBGs)^19^, and a multimode fiber^20^, have been proposed. Another recommended method to shift the range of amplification and the operating wavelength of the lasers is the amplified spontaneous emission (ASE) suppression method^21–25^. This method is made possible by suppressing the unwanted ASE spectrum for the improvement of gain of the transmission window. A S-band EDFA has been obtained by suppressing the C-band ASE using depressed cladding fiber (W fibers) with a variation of the refractive index^26^. In the case of TDFs, to achieve a fiber laser operating below 1800 nm with lasing extended to as short as 1710 nm, a piece of holmium-doped fiber (HDF) is placed between a two stage thulium-doped fiber amplifier (TDFA). The HDF acts as an absorber of wavelengths that are above 1800 nm and are from the ASE generated from the TDF during amplification^27^. In 2014, Yamada also demonstrated TDFL as a good candidate for gain amplification at 1700 nm^28^, when terbium is doped in the TDF cladding as the unwanted emission absorber. This condition contributes to the suppression of the 1800 to 2000 nm emission region. ASE Suppression based on photonic crystal fibers (PCFs) is the other novel method that has been proposed. In 2009, Varshney presented a single mode S-band erbium-doped PCF fiber amplifier based on concentric dual-core PCF structures. A complete suppression of ASE in the C-band region results in amplification gain in the S-band region^24^. For TDFA, to enhance the performance of a S-band fiber laser, thulium-doped photonic crystal fiber (TDPCF) was demonstrated^21^. The proposed method was based on 800 nm and 2000 nm band ASE suppression provided by the PCF’s unique geometric structure.

This paper reports an all-fiber tunable thulium-doped mode-locked laser with the two wide tunable ranges of 1702 to 1764 nm and 1788 to 1831 nm. This is achieved with a combination of partial ASE suppression and the nonlinear polarization rotation (NPR) technique. The ASE suppression is induced by a specially designed PCF in the laser cavity and indicates the extinction of the longer wavelengths after passing through the PCF. Two PCFs, namely, PCF (1) and PCF (2), are designed and applied to the mode-locked fiber laser setup to achieve desirable wavelength lasing.

## Results and Discussion

### PCF design for ASE suppression

**Figure 1:**
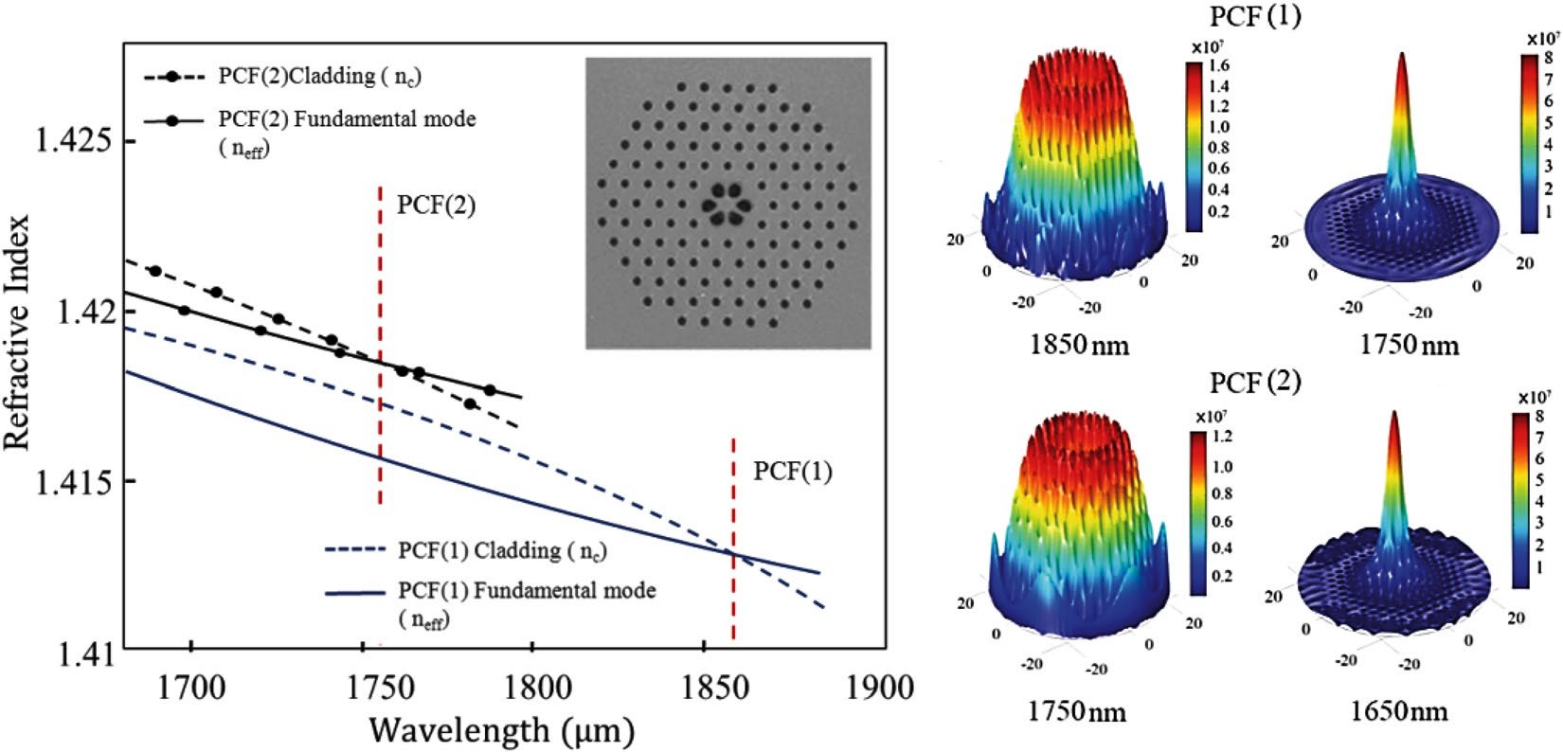
Suppression mechanism of ASE. (a) The variation of the fundamental mode and the cladding effective refractive index for PCF (1) and PCF (2). Inset: the electron microscope image of PCF (1). (b) Simulated E-field of PCF (1) and PCF (2) at different wavelengths.

Different structural specifications of PCFs lead to different transmission characteristics^29,30^. To design a PCF for filtering purposes the PCF’s structural characteristics are classified into four geometrical properties: lattice constant Λ, core diameter *D*, inner air hole ring diameter *d*′ that surrounds the core, and the outer ring air hole diameter *d*. It is important to highlight the parameters *d*′ and *d* since their ratio will influence the low-pass filter’s cut-off wavelength. In the proposed model, the air hole diameter in the inner ring is adjusted to achieve a long cut-off wavelength^31^. To reach the desired cut-off wavelength, *d*′/Λ is varied from 0.30 to 0.55 and *d*/Λ is varied from 0.25 to 0.40, in the simulation. The constants Λ and *D* for both of the PCFs fabricated in this study are similar and are equal to 3.2 *μ*m. PCF (1) has an inner hole ring diameter *d*′ of 2.5 *μ*m and an outer ring diameter *d* of 1.1 *μ*m while for PCF (2) the inner hole ring diameter *d*′ is 1.9 *μ*m and the outer ring diameter *d* is 0.7 *μ*m. The fabrication process is conducted in such a manner that the first layer of a different capillary diameter is followed by seven layers stacked together. Stacking is done hexagonally across a central rod which is fabricated using MCVD method. The inset of Fig. 1(a) shows the electron microscope image of the end face of PCF (1). The fabrication process exploited the classical stack and draw method, whereby the various capillary sizes of the first layer and the following seven layers were stacked hexagonally around a central rod that was fabricated via MCVD. Subsequent application of intra-air hole overpressure enabled control of the air hole dimensions in the final course of the drawing stage from the cane to the fiber.

**Figure 2:**
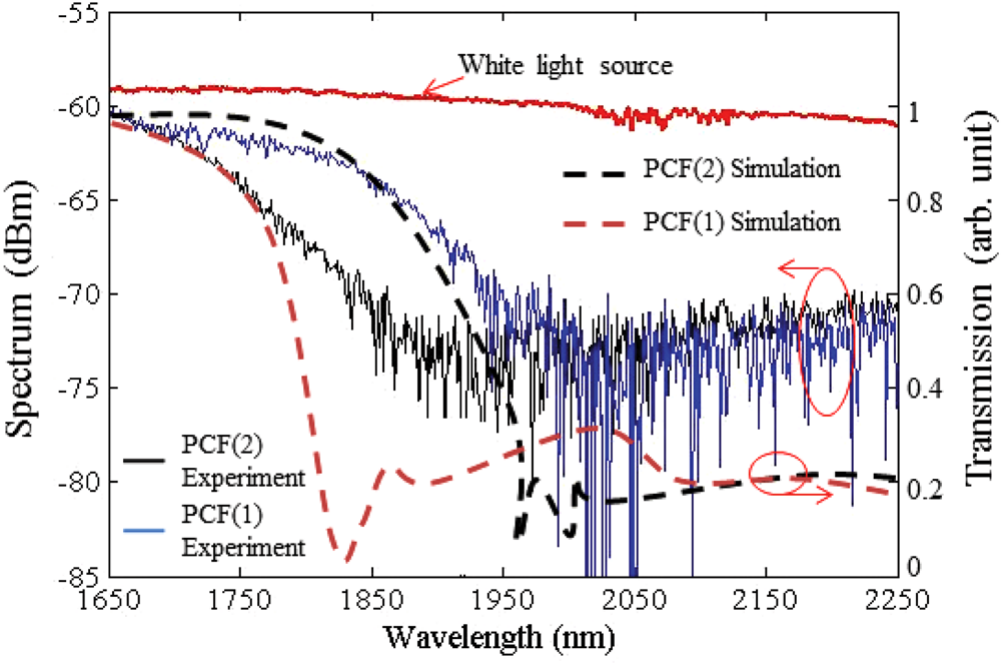
ASE and the transmission spectrum of the fabricated PCFs.

The graphs in Fig. 1(a) represent the refractive index of cladding and the fundamental mode of PCF (1) and PCF (2) as a function of wavelength. The intersection of these two parameters represents the long cut-off wavelength of the PCF. When the core effective refractive index is smaller than the cladding refractive index, the relationship between *d*′ and the core effective refractive index becomes noticeable at the corresponding wavelength. Therefore, when going beyond the long cut-off wavelength, the electromagnetic intensity moves toward the fiber cladding and hence contributes to a higher loss in the fiber. For PCF (1), the extinction occurs beyond 1850 nm while for PCF (2) it occurs beyond 1750 nm. Figure 1(b) shows the E-field graph of the PCF (1) at 1850 and 1750 nm and of the PCF (2) at 1750 and 1650 nm. This figure illustrates that at wavelengths shorter than the cut-off, the electric field operates in guided mode at the central core, while at wavelengths longer than the cut-off, the electric field again radiates inside the cladding region leading to higher loss. The experimental transmission spectral response of the PCFs and the results of modeled transmission spectra using the V-FEM method are shown in Fig. 2. The modeled transmission spectra comprise five rings of air holes are calculated based on the confinement loss^32^. To experimentally study the spectral response, 1.0 m of PCF is set up and a white light source with −58 dBm of power is injected into it. As observed from the fundamental mode and cladding effective refractive index variation graph in Fig. 1, there was a high loss of 15 dBm for wavelengths longer than 1850 nm for PCF (1) and 1750 nm for PCF (2). With reference to Fig. 2, a total of 1.68 dB of loss was measured at 1560 nm. Based on the total measured, the modeled PCF, and splice losses, the 0.65 dB extra loss is probably due to the overlapping losses between the PCF and SMF. It can be clearly seen that when using the same design parameters, both experimental and numerical results are in agreement.

### ASE suppressed TDFA

**Figure 3:**
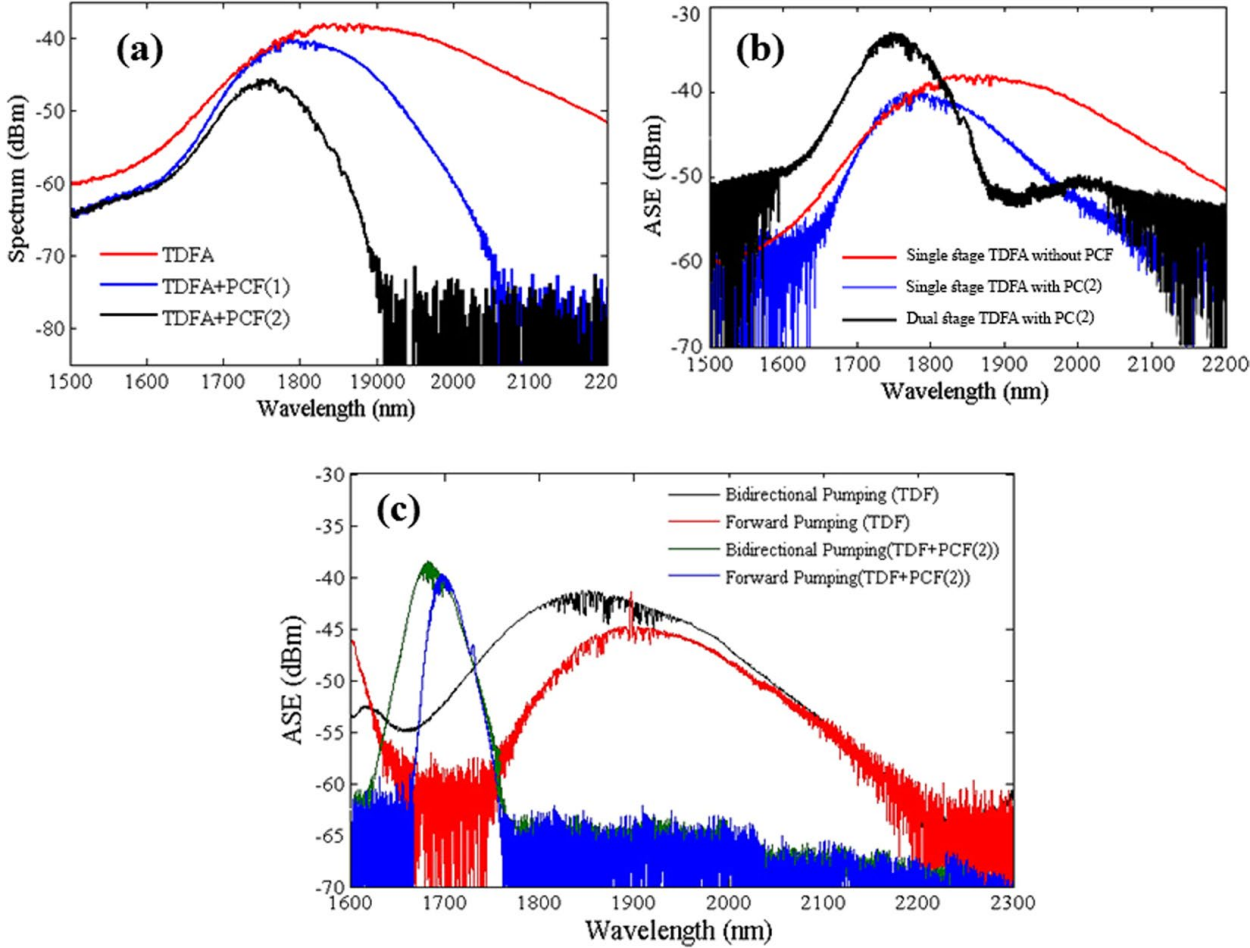
ASE spectrum of (**a**) single stage TDFA + PCF, (**b**) single and dual stages TDFA + PCF, and (**c**) dual stages TDFA and TDFA + PCF spectrum by bidirectional and forward pumping, under the same pumping power levels.

To elucidate the ASE suppression mechanism in a fiber laser, ASE suppression results of a TDFA are presented in this section. The configuration of the TDFA setup is presented in the methods section. The red curve in Fig. 3(a) shows the ASE spectrum of a standard single stage TDFA, while the blue and black curves represent the ASE spectrum of a TDFA for PCF (1) and PCF (2), respectively. In this case, the amplifier is forward pumped by a 788 nm laser with 181 mW of power. The generation of a broadband amplification region for the TDFA occurred at 1650–2100 nm. Figure 3(a) shows that there is a significant ASE suppression beyond the cut-off wavelength which is in agreement with the loss spectrum of Fig. 2. The amplification band of PCF (1) is shifted from 1700 to 1740 nm, and for PCF (2) from 1800 to 1820 nm due to the extinction of the longer wavelengths after passing through the PCF. To show the ASE suppression mechanism in a laser cavity, an experimental setup of a dual stage TDFA was completed, as shown in methods section. In the dual stage setup, the forward and the backward 788 nm laser power remained fixed at 100 mW at each stage. The ASE experimental results of the single stage TDFA without a PCF and the single and the dual stage TDFAs with PCF (2) are represented by red, blue and black curves, respectively, in Fig. 3(b). In comparison with the ASE spectrum of the single stage TDFA with a PCF (black curve in Fig. 3(b)), a higher ASE peak power at 1740 nm can be observed in the dual stage TDFA (black curve in Fig. 3(b)).

The broadening of the gain bandwidth is the main factor to achieve a wide tunable range. The bidirectional pumping method is adopted to widen the gain bandwidth of a TDF. Figure 3(c) shows an estimated gain bandwidth where the black and the red curves represent the bidirectional and the forward pumping schemes. It also shows ASE suppression of a TDF using PCF(2), where the green curve represents the bidirectional pumping, while the blue curve shows the forward one.

### ASE suppressed tunable mode-locked fiber laser

**Figure 4:**
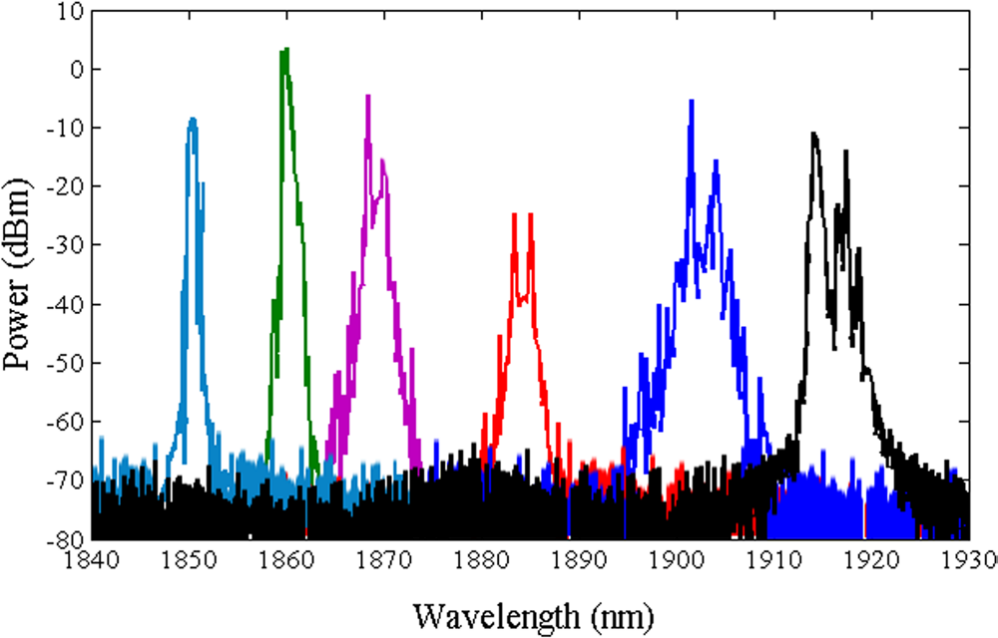
Tunable lasing emission of a non-ASE suppressed Tm-doped mode-locked all-fiber laser in the range of 1850–1920 nm enabled by the non-linear polarization rotation.

**Figure 5:**
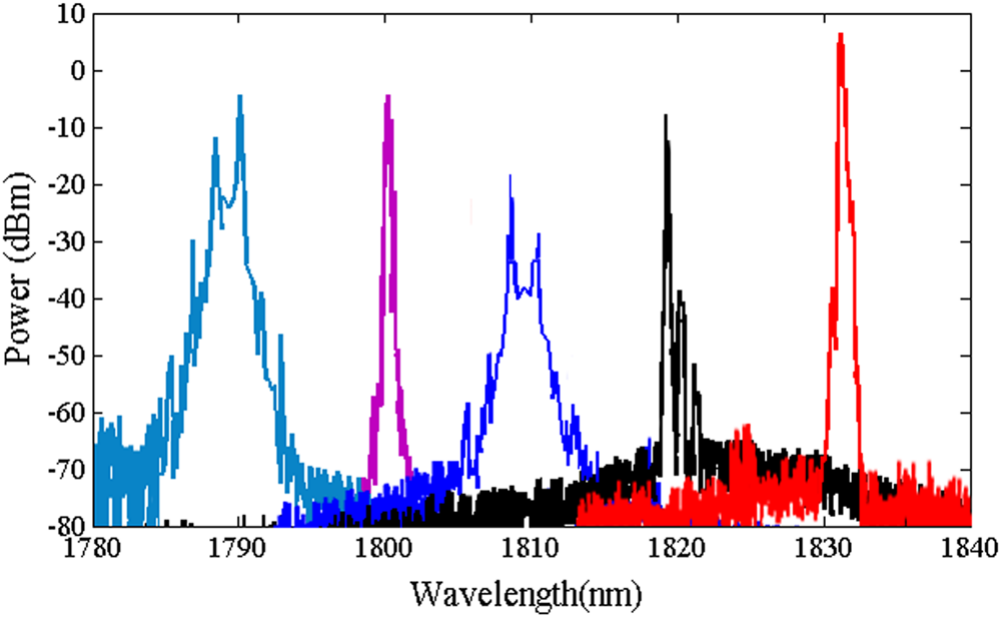
Tunable lasing emission of an ASE suppressed Tm-doped mode-lock all-fiber laser in the range of 1788–1831 nm enabled by PCF (1) and non-linear polarization rotation.

**Figure 6:**
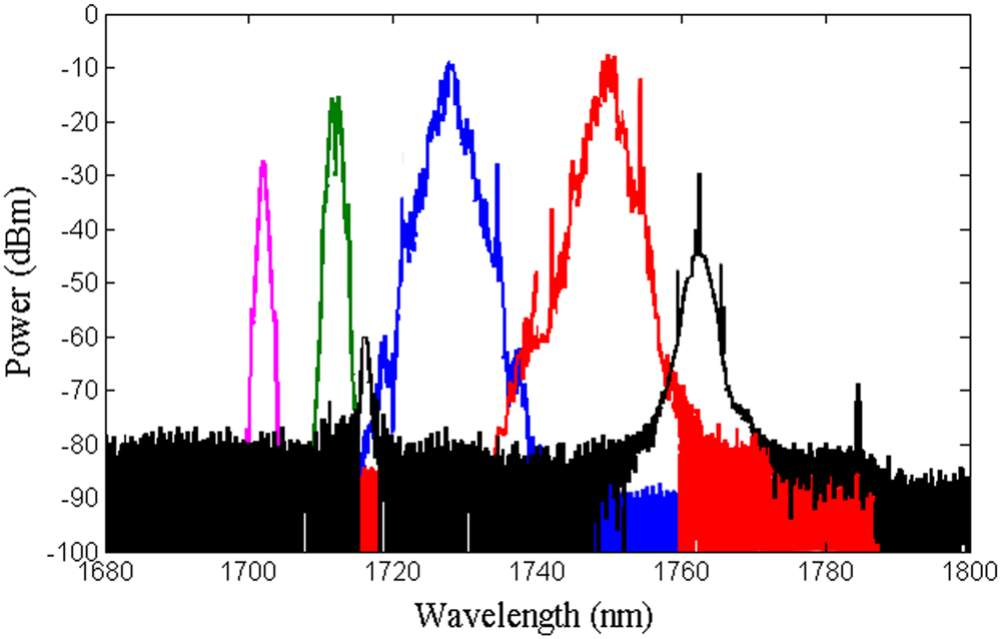
Tunable lasing emission of an ASE suppressed Tm-doped mode-lock all-fiber laser in the range of 1702–1764 nm enabled by PCF (2) and non-linear polarization rotation.

The output spectra of the mode-locked ring laser (see the methods section) developed with just TDFL, TDFL + PCF (1), and TDFL + PCF (2) are shown in Figs 4, 5 and 6, respectively. The power of the forward pump is set at 181 mW, while for the backward pump it is set at 255 mW. The soliton effect contributes to peak sidebands of the output pulse^32^, whereas water absorption contributes to the dip of the spectra^33^. The emission wavelength can be manipulated by tuning the polarization controller. The laser emits at a fixed peak position at any fixed state of the cavity polarization because the nonlinear polarization evolution prohibits the periodic spectral filters, which affect the entire range of the emission spectrum^34,35^.

Figure 4 shows the tunable lasing range of a normal TDFL without ASE suppression that falls in the 1848–1920 nm wavelength range. The 30 m PCF length in the cavity is used to shorten the periodicity of the modulated transmission, thus to increase the number of peaks in the wavelength range of laser emission. Figures 5 and 6 show that the tunable lasing range shifted to 1788–1831 nm and to 1702–1764 nm when PCF (1) and PCF (2) are added to the setup, respectively. The shift in the ASE peak power is because of the ASE suppression at longer wavelengths. Due to the 2–1 cross-relaxation process of the thulium ions, the conversion efficiency when lasing at 1800 nm is high. The relatively low efficiency observed at wavelengths lower than 1780 nm is due to the overlap between the emission and the absorption of the cross-sectional area, which leads to a low thulium inversion.

**Figure 7:**
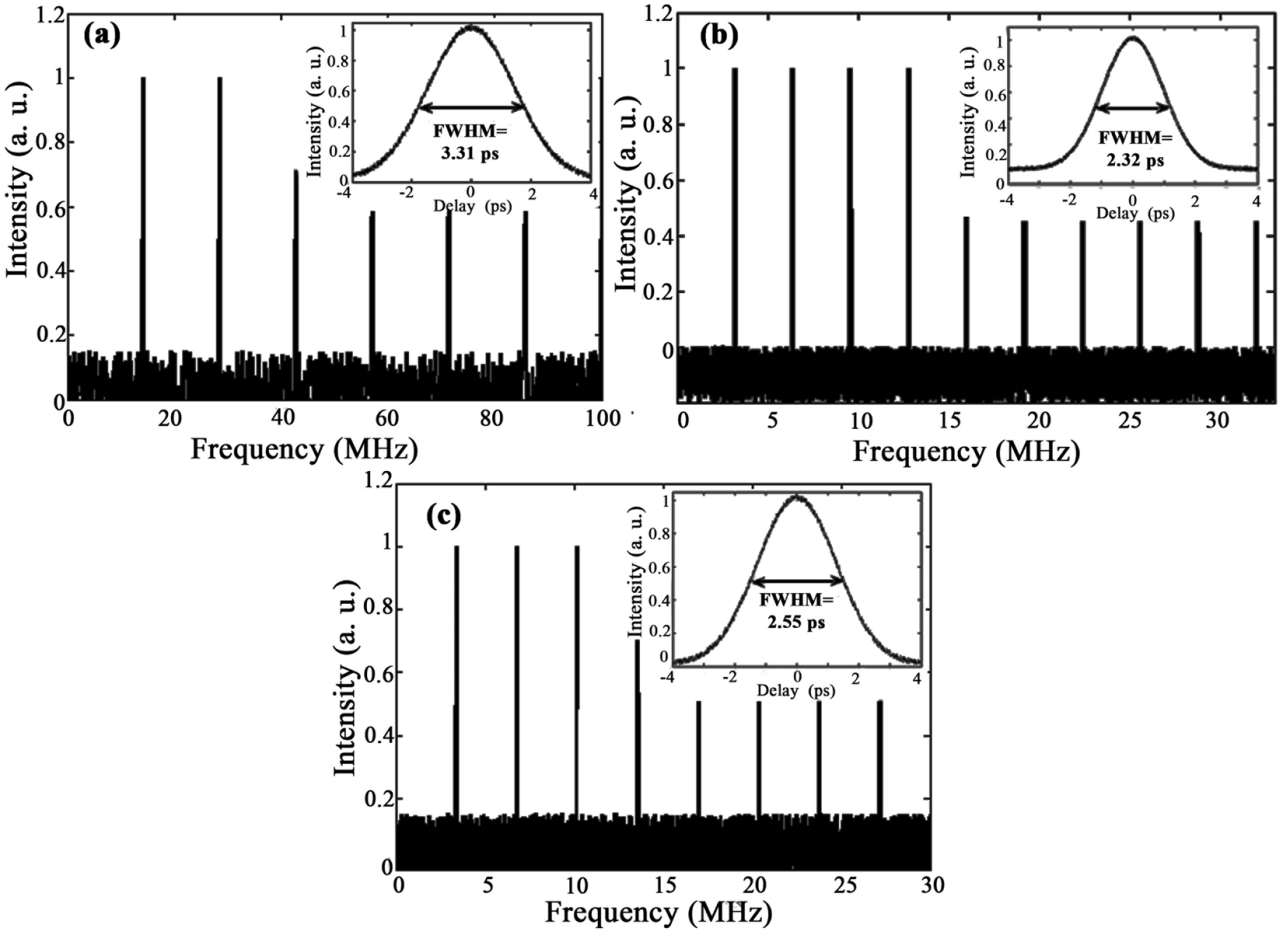
Pulse train and laser pulse width characteristic (inset) for (**a**) TDFL without a PCF at 1920 nm, (**b**)TDFL + PCF (1) at 1810 nm, (**c**) and TDFL + PCF (2) at 1730 nm.

A nominal pulse train of the laser for the TDFL without a PCF at 1920 nm, TDFL + PCF (1) at 1810 nm and TDFL + PCF (2) at 1730 nm is shown in Fig. 7(a–c), respectively. The autocorrelator trace of the laser pulse using the function fitting measurement is shown in the inset of the figures. For TDFL + PCF (1), the pulse full-width at half-maximum (FWHM) is measured to be 2.52 ps and the laser repetition rate in this case is 3.4 MHz. The measured pulse FWHM and the laser repetition rate for TDFL + PCF (2), is 2.55 ps and 3.2 MHz, respectively. A higher reputation rate of 14.3 MHz is observed for the TDFL without a PCF. This could be the result of the high length of the cavity related PCFs. For the TDFL without a PCF the pulse FWHM is approximately 3.31 ps.

## Conclusions

Two tunable ranges of a thulium doped mode-locked all-fiber laser are achieved. Based on the partial ASE suppression mechanism using two fabricated PCFs in conjunction with the NPR method, the emission band of the laser is shifted to shorter wavelengths. The intersection of the effective refractive index and the cladding refractive index for PCF (1) and PCF (2), which corresponds to the long cut-off wavelength, occurs at 1850 and 1750 nm, respectively. These intersections are due to the larger size of the air holes in the inner ring that surrounds the central core. For PCF (1), the tunable lasing range shifted to 1788–1831 nm, whereas for PCF (2) the emission spectrum shifted to the 1702–1764 nm range. The collected data could be further utilized in other simulations and experimental work for tunable thulium-doped mode-locked fiber lasers.

## Methods

### Suppressed band thulium-doped fiber optic amplifier system setup

**Figure 8:**
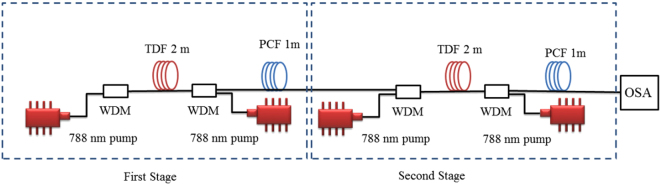
Configuration of the dual stage fiber optic amplifier with the first and second stages.

Figure 8 shows the configuration and measurement setup of a dual stage suppressed band thulium-doped fiber amplifier. The TDFA is characterized after the first stage and after the second stage. The architecture of the single stage TDFA consists of a TDF, a wavelength division multiplexing (WDM) coupler, a PCF, a pump laser and, an optical isolator. The TDF concentration is 2150 ppm with 27.00 dB/m of core absorption at the 788 nm wavelength. The numerical aperture (NA) and core diameter are 0.15 and 9.0 *μ*m, respectively. The TDF length is optimized at 2.0 m while the PCF length is fixed at 1.0 m. The WDM coupler is used to combine the input signal with the pump. The optical isolator is placed after the signal to ensure a unidirectional operation of the optical amplifier. Two continuous wave (CW) tunable titanium-sapphire lasers are used as the pumps for the forward and backward pumping configurations. The operational wavelength of the laser is fixed at the TDF peak absorption of 788 nm. A Yokogawa AQ6375 optical spectrum analyzer (OSA) is used to measure the amplified spontaneous emission (ASE) spectrum.

### Tunable mode-locked fiber laser system setup

**Figure 9:**
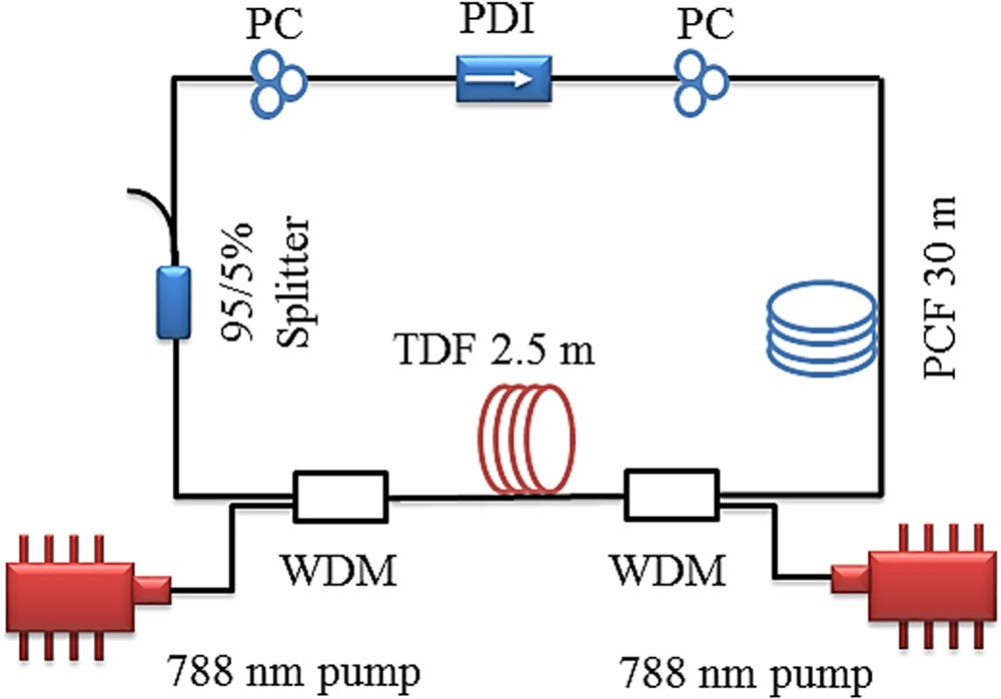
Schematic of the tunable mode-locked fiber laser system setup.

The system setup for the tunable mode-locked fiber laser is shown in Fig. 9. This setup consists of an optimized 2.5 m of the same TDF as the amplifiers. The TDF is bi-directionally pumped by two CW tunable titanium-sapphire lasers operating at 788 nm via two WDM couplers. The polarization dependent isolator sandwiched between the two polarization controllers serves as the mode locker, which induces the NPR mechanism. At the output 95:5 coupler, 5% of the power is channeled as the cavity output and the rest propagates in the cavity. A long length of PCF (30 m) in the cavity is used to shorten the periodicity of the modulated transmission, thus increasing the number of peaks in the wavelength range of laser emission. However, a longer PCF would lead to more transmission loss and affect the mode-locking threshold. The dispersion coefficients of the TDF and the PCF at 1790 nm are −72 and approximately −65.2 *ps* ^2^/*km*, respectively.
